# An Active Electromagnetically Induced Transparency (EIT) Metamaterial Based on Conductive Coupling

**DOI:** 10.3390/ma15207371

**Published:** 2022-10-21

**Authors:** Wu Zhang, Jiahan Lin, Xiaohui Fang, Yanxiao Lin, Kai Wang, Meng Zhang

**Affiliations:** 1 School of Physics and Material Science, Guangzhou University, Guangzhou 510006, China; 2 Department of Electrical and Electronic Engineering, Southern University of Science and Technology, Shenzhen 518055, China; 3 School of Electronics and Communication Engineering, Guangzhou University, Guangzhou 510006, China

**Keywords:** metamaterial, electromagnetically induced transparency, tunable

## Abstract

In this paper, we demonstrate an active metamaterial manifesting electromagnetically induced transparency (EIT) effect in the microwave regime. The metamaterial unit cell consists of a double-cross structure, between which a varactor diode is integrated. The capacitance of the diode is controlled by a reversed electrical bias voltage supplied through two connected strip lines. The diode behaves as a radiative resonant mode and the strip lines as a non-radiative resonant mode. The two modes destructively interference with each other through conductive coupling, which leads to a transmission peak in EIT effect. Through electrical control of the diode capacitance, the transmission peak frequency is shifted from 7.4 GHz to 8.7 GHz, and the peak-to-dip ratio is tuned from 1.02 to 1.66, demonstrating a significant tunability.

## 1. Introduction

Electromagnetically induced transparency (EIT) is a quantum effect that stems from the destructive interference in atomic systems [1,2]. In EIT effect, an absorptive resonance is coupled to a non-absorptive resonance, which results in a narrow transparent spectrum window and can realize a high Q-factor transmission peak. Although first reported in the quantum optics field, EIT effect was later demonstrated in other optical systems such as photonic crystals [3], resonator systems consisting of chiral optical states [4] and metamaterials [5,6]. Metamaterial is an artificially designed subwavelength unit cell array and used for manipulating electromagnetic waves. The unit cell can be flexibly designed and induces the desired resonant mode at different EM spectrum bands depending on the unit cell structure and coupling within the unit cell [7]. Therefore, metamaterial has the flexibility advantage for the electromagnetic (EM) wave control, and has demonstrated wide applications such as polarization rotation [8], absorption [9], imaging [10], detection [11], information coding [12], etc.

In the unit cell of metamaterial for the EIT effect, a bright resonator and a dark resonator in close proximity are designed and coupled with each other, resulting in an extraordinary transparency and strong dispersion. In 2008, N.I. Zheludev’s group reported a classical EIT-like effect in a planar metamaterial which generates a considerable delay of propagating pulses with propagation distance much smaller than the wavelength [13]. The same group later also demonstrated that the resonant transmission frequency in the EIT-like metamaterial can be polarization-independent and incident-angle-independent [14]. The EIT metamaterial since then has been demonstrated in different frequency regimes from the microwave regime [15] to the terahertz regime [16,17] and the optical regime [18]. Due to the successful realization of the EIT effect in the metamaterial, various applications were demonstrated such as sensing [19,20] and slow light [21,22]. While in the metamaterial the resonant mode is usually within a narrow band, EIT effect was also usually limited in a narrow spectral range. To realize a broadband EIT effect in the metamaterial, different resonance tunable approaches have been proposed [23]. For example, Micro-Electro-Mechanical System (MEMS) technology is applied to mechanically reconfigure the micrometer scaled metamaterial unit cell structure and change the coupling between the radiative mode and the non-radiative mode [24,25]. In addition, different intrinsic property tunable materials such as vanadium dioxide (VO_2_) [26,27,28], graphene [29,30,31] and superconductors [32,33] were utilized to construct the EIT metamaterial which is tuned under external stimulus. Another promising strategy is to integrate an electrical diode in the EIT metamaterial unit cell which is manipulated through the electrical bias on the diode. For example, PIN diodes were used in the EIT metamaterial, the resistance of which can be tuned through electrical bias, resulting in a change in the resonance transmission [34,35]. The control on PIN diodes usually only changes the resonance transmittance at a fixed resonant frequency. On the other hand, the varactor diode has an up to 1 GHz high operating frequency and the change of its capacitance will shift the resonant frequency in the metamaterial [36]. The capacitance of the varactor diode is changed under different bias voltages, which realizes a tunable transmission peak frequency in the EIT metamaterial. In such metamaterial designs, the strip lines supplying voltage bias to the diode will usually affect the resonance in the metamaterial; therefore, most designs only used a single unit cell for EIT effect realization, and only worked at relatively low frequency range to avoid the effect from the strip lines [37,38]. Here, we designed a tunable EIT metamaterial in which the varactor diode serves as the radiative mode, and the strip lines as the non-radiative mode. Therefore, the strip lines will contribute to the EIT effect instead of affecting the performance. The EIT metamaterial is designed as an array of unit cells and through the electrical control, a significant frequency shift is experimentally demonstrated for the transparency window, and the transmittivity is also flexibly tuned in real time.

## 2. Materials and Methods

**Figure 1:**
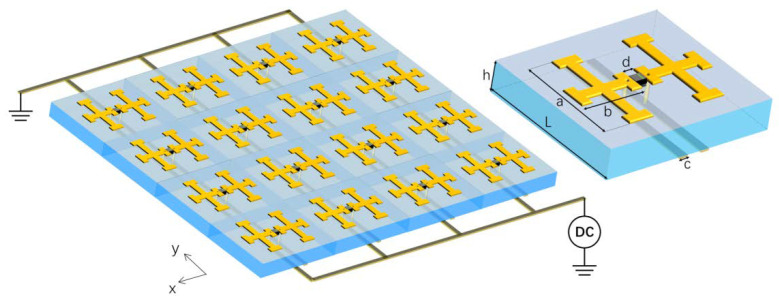
The schematic of the tunable EIT-like metamaterial; the insertion: the metamaterial unit cell.

The designed EIT metamaterial consists of a planar array of unit cells with double-cross structures as shown in Figure 1. A varactor diode is integrated between the double cross along the *x*-direction and two strip lines are patterned on the backside of the substrate along the *y*-direction. The two strip lines connect to the two ends of the varactor diode through two via holes. A reversed biased voltage is applied to the varactor diode through the two strip lines and tunes the capacitance of the varactor at different voltages. The dimensions of the metamaterial unit cell are as shown in the insertion of Figure 1. The lattice constant of the metamaterial unit cell *L* is 7 mm × 7 mm and the substrate thickness *h* is 1.6 mm. The length *a* and width *b* of the cross structure are 5 mm and 3.6 mm, respectively. The width of the strip line *c* is set at 0.5 mm, and the distance between the two cross structures *d* is 1.3 mm. The thickness of the crosses and strip lines are 35 μm. With a x-polarized planar EM wave incidence, resonances along the short arms of the two cross structures and on the varactor diode are expected to be induced, which behaves as a radiative mode. The two strip lines are perpendicular to the incident electrical field and behave as a non-radiative mode. The above two modes conductively couple with each other through the via hole in the substrate and induce EIT effect, which is elaborated in the next section.

## 3. Results

**Figure 2:**
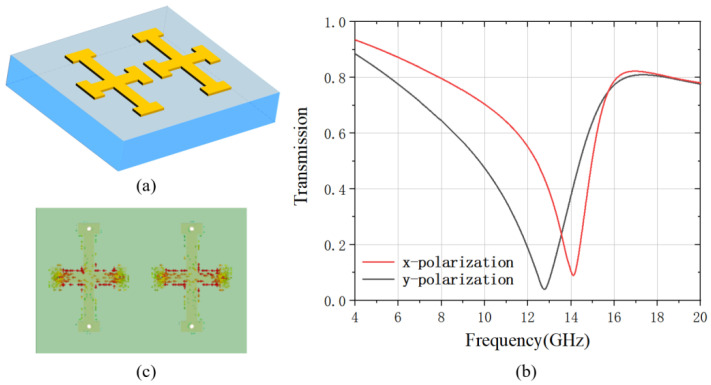
(**a**) the schematic of the double-cross unit cell; (**b**) the simulated x-polarized and y-polarized transmission of the double-cross metamaterial; (**c**) the surface current of the x-polarization dip at 14.1 GHz.

First, we analyzed the transmission of the metamaterial only consisting of the double-cross unit cell without the varactor diode and strip lines as illustrated in Figure 2a. The metamaterial is modeled using Microwave Studio of Computer Simulation Technology (CST) in periodic boundary condition. The substrate of the metamaterial is made of FR4 with permittivity of 4.3, and the double-cross and strip line are made of copper with conductance of 5.8 × 10^7^ S/m. A linearly polarized plane wave is normally incident on the metamaterial along the z-direction. The transmission spectra of the metamaterial from 4 GHz to 20 GHz are numerically analyzed as shown in Figure 2b. A significant resonant dip is observed at 14 GHz and 12.8 GHz for the x-polarized and the y-polarized transmission, respectively. The resonance under the x-polarization is analyzed by calculating the surface current as shown in Figure 2c, which indicates that the resonant dip is a simple dipole mode.

**Figure 3:**
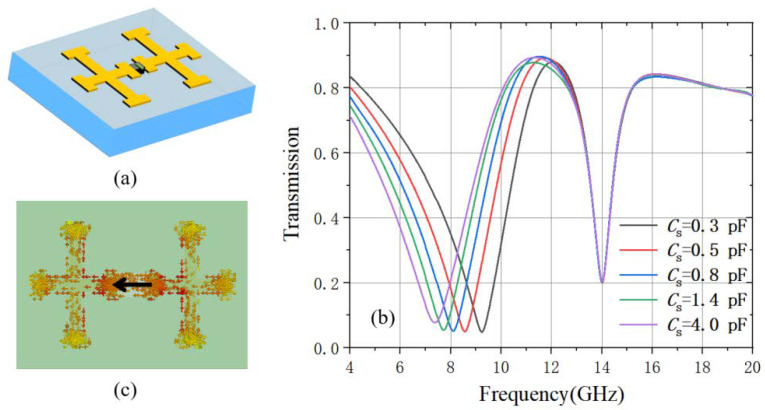
(**a**) the schematic of the double-cross and varactor diode unit cell and (**b**) its x-polarized transmission at different varactor diode capacitance and (**c**) its surface current of the transmission dip at 7.8 GHz when the diode capacitance is 1.4 pF.

Then the varactor diode is integrated in the unit cell model between the double-cross structure as shown in Figure 3a. The varactor diode is modeled as an RLC serial circuit, in which the resistance is set at Rs = 2.5 Ω and inductance Ls = 0.7 nH, while the capacitance Cs is a tunable value [39]. We first simulated the x-polarized transmission spectrum of the metamaterial as shown in Figure 3b. The dip resonance at 14 GHz is the same as that in the double-cross metamaterial, while a new dip resonance is raised in the frequency regime from 9.2 GHz to 7.6 GHz in the x-polarized transmission spectrum by tuning *C*_s_ from 0.3 pF to 4.0 pF. The transmission at the dip is below 10%. The decreases of the resonant frequency with the increasing diode capacitance can be explained by the relation between the resonance and the LC circuit. By investigating the surface current at the frequency dip *f* = 7.7 GHz when the varactor diode capacitance is 1.4 pF under the x-polarized incidence as shown in Figure 3c, it can be seen that the surface current focuses on the diode structure and flows in the *x*-direction. The black arrow indicates the instantaneous current flow direction. Here we make use of this resonance as the lossy radiative mode, the frequency of which is tunable depending on the varactor diode capacitance *C*_s_. It is also worth pointing out that the resonance at a higher frequency of 14 GHz does not shift during the varactor diode capacitance change. This is because the resonance is on the short horizontal arms of the double cross structure and only depends on the arm length. As this resonance does not contribute to the EIT effect of the metamaterial, we will skip discussing it in the following part.

**Figure 4:**
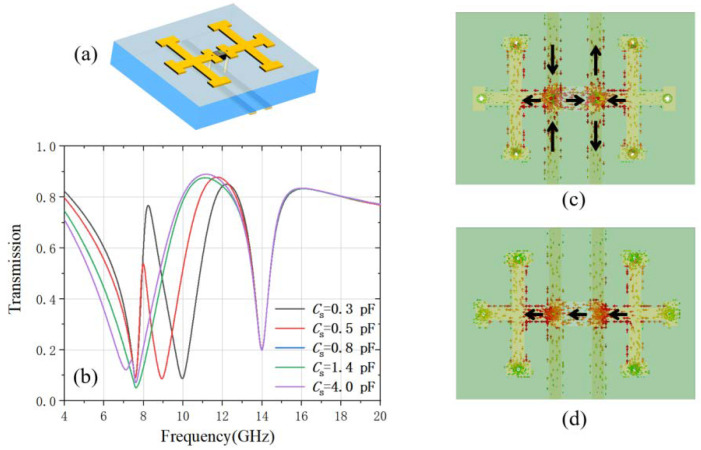
(**a**) the schematic of the double-cross, varactor diode and strip line unit cell; (**b**) its x-polarized transmission at different varactor diode capacitance; (**c**) the surface current of the transmission peak at 8.5 GHz when the diode capacitance is 0.3 pF and (**d**) the surface current of the transmission peak at 7.6 GHz when the diode capacitance is 1.4 pF.

Then the two strip lines are added at the backside of the substrate and connect to the two ends of the varactor diode through the via holes in the substrate as shown in Figure 4a. The two strip lines not only connect to the electrical bias for experiment testing, but also serve as the non-radiative mode and conductively couple to the radiative mode induced on the varactor diode. The interference between the two modes can be expressed by two coupled differential equations [40]:

(1) $$\{\begin{array}{c} {\ddot{x}}_{1}+\gamma_{1}{\dot{x}}_{1}+\omega_{1}^{2}x_{1}+\Omega x_{2}=gE_{0}e^{j\omega t}\\ {\ddot{x}}_{2}+\gamma_{2}{\dot{x}}_{2}+\omega_{2}^{2}x_{2}+\Omega x_{2}=0 \end{array}$$

where *x*_i_, *γ*_i_ and *ω*_i_ are the magnitude, the loss and the resonant frequency of the radiative mode for *i* = 1 and the non-radiative mode for *i* = 2. Ω is the coupling coefficient between the two resonant modes, while g presents the coupling strength of the radiative mode resonator with the incident electrical field *E*_0_*e^jωt^*. According to the two coupled equations, the susceptibility of the EIT metamaterial can be derived as

(2) $$\chi =\chi_{r}+i\chi_{i}\propto \frac{\omega -\omega_{2}+\frac{i\gamma_{2}}{2}}{\left(\omega -\omega_{1}+\frac{i\gamma_{1}}{2}\right)\left(\omega -\omega_{2}+\frac{i\gamma_{2}}{2}\right)-\frac{\Omega^{2}}{4}}$$

As the energy loss is proportional to the imaginary part of χ, the transmittance of the system *T*$\propto 1-i\chi_{i}$. Furthermore, as *ω*_1_ is the resonant frequency of the radiative mode induced on the varactor diode between the two cross structures, it has the relation *ω*_1_ $\propto \frac{1}{\sqrt{\mathrm{LC}}}$, where *L* and *C* are the effective inductance and capacitance of the radiative mode. *C* is mainly contributed from the varactor diode capacitance *C*_s_. The increase of *C*_s_ will cause the red shift of *ω*_1_.

The transmission of the metamaterial is numerically calculated under the x-polarized incidence. A transmission peak is observed within the dip resonance regime caused by the varactor diode as shown in Figure 4b. The transmittance and the frequency of the transmission peak can be tuned by changing the varactor diode capacitance. When the varactor diode capacitance is 0.3 pF, the transmission peak is at 8.5 GHz with 75% transmittance. Further investigating the surface current at this transmission peak, as shown in Figure 4c, strong surface current is observed on the two strip lines along the y-direction even under the x-polarized incidence. The black arrows indicate the instantaneous flow direction of the surface current, which flows in opposite directions on the two strip lines. This is caused by the conductive coupling with the varactor diode and the double-cross structures. The strip lines now behave as the non-radiative mode. Meanwhile, the surface current on the varactor diode significantly decreases compared with that in Figure 3c in which there is no coupling with strip lines. Therefore, we can conclude that the conductive coupling between the varactor diode and the strip lines induces the EIT effect. As the varactor diode capacitance increases, the radiative mode frequency *ω*_1_ decreases. The frequency of the transmission peak also experiences a red shift and the corresponding transmittance gradually deceases. As the diode capacitance increases to 1.4 pF, the transmission peak is totally suppressed and only one resonant dip at 7.6 GHz is observed. The surface current at this frequency is shown in Figure 4d. The surface current is mainly on the varactor diode and on the short beams of the two cross structures close to the diode. The currents are all along the x-direction and therefore only radiative mode is excited, which explains that only resonant dip is observed at this diode capacitance.

**Figure 5:**
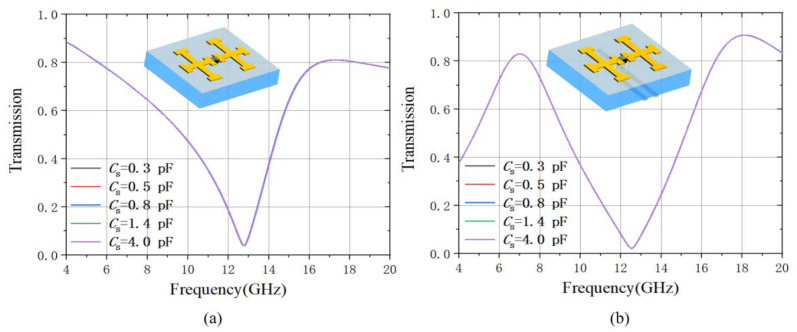
(**a**) the y-polarized transmission of the metamaterial with double-cross and diode unit cell; (**b**) the y-polarized transmission of the metamaterial with double-cross, diode and strip lines unit cell.

We also investigated the y-polarized transmission spectra of the metamaterial when the unit cell is without and with the strip lines as shown in Figure 5a,b, respectively. The transmission spectrum in Figure 5a is almost the same as that of the metamaterial consisting of double-cross structure without the varactor diode, regardless of the value of varactor diode capacitance. Therefore, as can be expected and shown in Figure 5b, the y-polarized transmission of the metamaterial with strip lines only manifests a new resonance raised from the strip lines, which is independent of the varactor diode. Therefore, this EIT effect is polarization-sensitive and is only excited in the x-polarized incidence.

**Figure 6:**
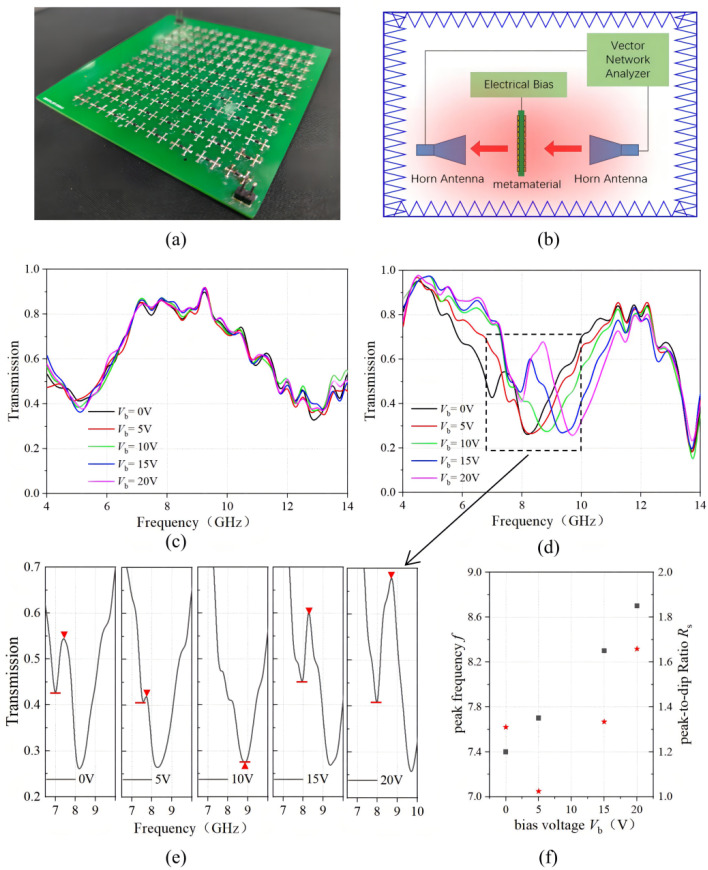
(**a**) the experiment set up illustration; (**b**) the fabricated metamaterial sample consisting of double-cross, varactor diode and strip line structures; (**c**) the y-polarized transmission and (**d**) the x-polarized transmission of the metamaterial at different *V*_b_; (**e**) the change of the transmission peak (red triangle: peak frequency; short bar: dip frequency); (**f**) the peak frequency and peak-to-dip ratio at different *V*_b_ (black square: peak frequency; red start: peak to dip ratio).

The metamaterial is fabricated using the standard double layer PCB technology. As shown in Figure 6a, the metamaterial has 8 × 12 unit cells in the *x*–*y* direction. The double-cross structure is patterned on the top of the PCB board and the two strip lines are patterned at the bottom. The varactor diode (SMV2020, Skyworks, Irvine, CA, USA) is soldered between the double-cross structure. A reversed bias voltage *V*_b_ was applied between the varactor diode by connecting the anode and the cathode to the corresponding strip line at the backside. Increasing the voltage from 0 V to 20 V, the capacitance of the varactor diode decreased from 3.2 pF to 0.35 pF according to the diode data sheet as shown in Table 1 below.

The transmission of the metamaterial was measured using a vector network analyzer and the experimental setup is illustrated in Figure 6b. For the y-polarization incidence, the transmission spectrum is a Fabry–Perot envelope, which is due to the resonance between the substrate as shown in Figure 6c. Therefore, the spectrum does not change with the bias voltage change. For the x-polarization incidence, a transmission dip is observed at around 9 GHz, while a transparency window is induced in the dip depending on the applied reversed bias voltage as shown in Figure 6d. When *V*_b_ = 10 V, only the transmission dip is obtained with a transmittance of 27%, which corresponds to a diode capacitance of 0.57 pF. As the reversed bias voltage decreases to 5 V and 0 V, the diode capacitance increases to 1.19 pF and 3.20 pF, and a transmission peak appears at 7.7 GHz and 7.4 GHz, respectively. While as the revised bias voltage increases to 15 V and 20 V, the varactor diode capacitance changes to 0.41 pF and 0.35 pF, and the transmission peak shifts to 8.28 GHz and 8.73 GHz, respectively. Compared with Figure 4c, the transmission window and the tuning of the transmission peak of the experimental results matches well with the simulation results. The transmittance and the Q-factor of the transmission peak, however, do not fully agree with the simulation results, which should be due to the limited size of the fabricated sample and the loss during the wave propagation. The experimental tunability of EIT is further quantified by evaluating the transmission peak frequency *f*, indicated by the red triangles in Figure 6e, and peak-to-dip ratio *R*_s_, or the ratio of the peak transmittance to the closest dip transmittance, indicated by the short bars in Figure 6e for each reversed bias voltage. The transmission peak frequency is tuned from 7.4 GHz to 8.7 GHz with the increases of the *V*_b_ from 0 V to 20 V, while the peak-to-dip ratio *R*_s_ is tuned from 1.31 to 1.02 when *V*_b_ increases from 0 V to 5 V, and tune from 1.33 to 1.66 when *V*_b_ increases from 15 V to 20 V, which are denoted as the black square and the red star as shown in Figure 6f, respectively. Therefore, EIT effect can be significantly tailored through the electrical control.

We then compared the measurement results with previous diode based EIT metamaterial as shown in Table 2. It can be seen that the PIN diode based EIT metamaterial only changes the transmittance of the transmission peak [34,35], while for the varactor diode based EIT metamaterial, previous designs only consisted of a single unit cell with a transmission peak relatively low frequency regimes (less than 4 GHz) [37,38]. In this work, instead, a much higher resonant peak above 7 GHz frequency was observed. In addition, we define the relative tuning bandwidth ratio (RTBR) *r*_T_, or the ratio of the tuned frequency Δf to the central frequency $f_{c}$ to describe the tunability of the EIT metamaterial. We can see *r*_T_ is relatively small (less than 10%) for the previous works listed in Table 2; however, in this work, a much higher *r*_T_ up to 16.1% was observed.

## 4. Conclusions

In conclusion, we demonstrated a tunable EIT effect in the microwave regime by conductively coupling a radiative mode and a non-radiative mode in the metamaterial. A varactor diode was utilized to serve as the radiative mode, which is tuned through electrical voltage bias. The strip lines connected to the voltage supply served as the non-radiative mode and conductively coupled with the varactor diode. The transmission peak of the EIT was shifted from 7.4 GHz to 8.7 GHz with a relative tuning bandwidth ratio of 16.1%, and the peak-to-dip ratio can be tuned from 1.02 to 1.66, demonstrating a significantly tunable EIT effect in the metamaterial. In addition, the use of strip lines as the non-radiative mode provides a more convenient approach for the EIT metamaterial design.

## Figures and Tables

**Table 1:** Relation between the applied voltage and capacitance of the varactor diode.

bias Voltage	0 V	5 V	10 V	15 V	20 V
capacitance	3.20 pF	1.19 pF	0.57 pF	0.41 pF	0.35 pF

**Table 2:** EIT metamaterial performance literature comparison.

References	Diode Type	Single Cell/Array	Transmittance Change	Peak Frequency (GHz)	$r_{T}=\frac{\Delta f}{f_{c}}$
[34]	PIN	single unit cell	Yes	5.25	0
[35]	PIN	unit cell array	Yes	6.28	0
[37]	varactor	single unit cell	Yes	1.41~1.54	8.7%
[38]	varactor	single unit cell	Yes	3.04~3.26	7.0%
our work	varactor	unit cell array	Yes	7.4~8.7	16.1%

## Data Availability

Raw data presented in this study are available on request from the corresponding author.
